# A systematic review on the efficacy of virtual reality and gamification interventions for managing anxiety and depression

**DOI:** 10.3389/fdgth.2023.1239435

**Published:** 2023-11-07

**Authors:** Nuru Jingili, Solomon Sunday Oyelere, Markus B. T. Nyström, Lina Anyshchenko

**Affiliations:** ^1^ Department of Computer Science, Electrical and Space Engineering, Luleå University of Technology, Skellefteå, Sweden; ^2^ Department of Health, Education and Technology Division, Luleå University of Technology, Luleå, Sweden

**Keywords:** anxiety, depression, virtual reality, randomized controlled trials, mental health

## Abstract

This systematic review aims to assess the effectiveness of virtual reality (VR) and gamification interventions in addressing anxiety and depression. The review also seeks to identify gaps in the current VR treatment landscape and provide guidelines for future research and development. A systematic literature search was conducted using Scopus, Web of Science, and PubMed databases, focusing on studies that utilized VR and gamification technology to address anxiety and depression disorders. A total of 2,664 studies were initially identified, 15 of those studies fulfilled the inclusion criteria for this systematic review. The efficacy of VR in addressing anxiety and depression was evident across all included studies. However, the diversity among VR interventions highlights the need for further investigation. It is advised to incorporate more diverse participant samples and larger cohorts and explore a broader spectrum of therapeutic approaches within VR interventions for addressing anxiety and depression to enhance the credibility of future research. Additionally, conducting studies in varying socioeconomic contexts would contribute to a more comprehensive understanding of their real-world applicability.

## 1. Introduction

In recent years, the prevalence of anxiety and depression disorders has surged, posing a significant global health challenge (1). These debilitating conditions not only compromise the well-being of individuals but also place a substantial burden on healthcare systems and society at large (1). In the quest for innovative and effective interventions, virtual reality (VR) and gamification have emerged as promising tools that harness technology to address mental health concerns. This systematic review examines and analyses the efficacy of virtual reality and gamification interventions in managing anxiety and depression.

Gamification is essentially the art of applying elements commonly found in game design to non-game scenarios (2). It's as if you take the best aspects of games and seamlessly integrate them into more serious environments. This integration can include elements like earning points, advancing through levels, competing on leaderboards, and proudly displaying digital badges (3). Gamification has gained increasing prominence in recent years as a tool to enhance engagement, motivation, and behavior change in various domains, including mental health (4). Persuasive technology is the art of influencing attitudes and behaviours without coercion or deceit (5). These persuasive features often tap into cognitive and emotional mechanisms, harnessing the power of feedback, social influence, and competition to promote positive change (6). The persuasive features inherent to gamified interventions leverage psychological principles to encourage individuals to adopt desired behaviours, sustain engagement, and achieve positive outcomes.

Virtual Reality is an innovative technological interface that allows users to immerse themselves in computer-generated environments within a controlled setting. With its increasing utilization in mental health treatment and clinical research, VR has emerged as a promising technological intervention in the field. The primary goal of VR is to create a simulated world that closely parallels reality, offering an immersive and interactive experience for users. To achieve this, various technological elements such as head-mounted displays (7–10), eye tracking devices (11), synthesized sounds (12), and motion sensing devices are employed (13). These components enable users to engage their senses and actively explore the virtual environment, facilitating a sense of presence and immersion. Furthermore, some VR applications respond to the user's actions, allowing for dynamic interaction and a more naturalistic experience. The controlled nature of VR environments provides a safe space for consistent replication, testing, and modification without compromising real-world applicability. By using a head-mounted display (HMD) and immersive 3D environments, VR enables users to experience realistic and controlled scenarios that trigger responses and facilitate therapeutic outcomes (14–16).

The use of VR in mental health has garnered significant attention as a potential tool for preventing and treating mental illness while promoting mental well-being (17). Advancements in mobile and commercial VR capabilities have made it more feasible and affordable to implement this technology in mental health interventions (18). VR interventions have shown promise in addressing anxiety and depression, providing users with safe and immersive environments to learn coping strategies and manage their conditions (19). Anxiety and depression are two common mental health disorders that can significantly impact individuals' quality of life. VR interventions have emerged as an innovative approach to augment traditional treatment methods. VR for treating anxiety and depression has gained interest due to its innovative potential in treatment. VR has the potential to provide users with safe, non-threatening environments where patients can experience a different world and learn how to cope with their anxiety and depression (15).

Numerous studies have investigated the effectiveness of VR interventions for anxiety and depression disorders. A recent systematic review of 721 studies highlighted exposure therapy as the most researched topic, with substantial evidence supporting its efficacy (16). In the context of anxiety, VR has demonstrated the ability to elicit realistic responses to feared stimuli, facilitating exposure-based therapies (18). However, while there is a growing body of research on VR's effectiveness in anxiety disorders, the evidence for its use in depression is less conclusive, and further investigation is needed to bridge this gap (16, 18, 19).

Incorporating gamification and persuasive technology into VR interventions have great potential to keep users engaged and motivated to adhere to the treatment of anxiety and depression. By incorporating elements of play, competition, and rewards, gamification can motivate individuals to engage in activities that alleviate symptoms of anxiety and depression. When applied to VR Interventions, persuasive technology can provide tailored interventions, increase engagement, provide personalized support, and empower individuals to take an active role in their well-being. Moreover, VR has the potential to contribute to accessible and cost-effective mental healthcare services.

When remote and self-management attributes are embedded within VR interventions, it can diminish or eliminate the necessity for direct therapist involvement throughout the intervention process. These features enable individuals to assume a greater degree of self-reliance in managing their mental health conditions. Research unequivocally demonstrates the feasibility of integrating self-management features within VR interventions, ultimately empowering individuals to take charge of their treatment journey and interact with the intervention at their own pace and convenience (20, 21). VR interventions are a promising solution due to their versatile and easily accessible nature for remote settings where in-person therapy may pose accessibility challenges or logistical impracticalities. VR remote and self-management capabilities allow for the delivery of interventions outside traditional clinical settings, enhancing convenience and flexibility for patients. Some ongoing or planned studies explore the potential of administering mental health treatment remotely through VR (22, 23). However, a notable gap in current research pertains to the inconclusive evidence of its efficacy, given that certain studies have involved researchers directly during interventions or not yet finished (7, 8, 22). By exploring the multifaceted nature of VR and integrating gamification, this review establishes the foundation for comprehending how VR, with its diverse modalities, can be leveraged to optimize remote work settings. Such optimization can enhance overall productivity, increase patient convenience and employee well-being, and promote a more seamless and effective integration of remote work into daily routines and can be scaled and distributed to a wide range of users without significant increases in provisions and maintenance costs (12, 16, 19, 24).

Despite the growing interest in VR for anxiety and depression, there is a scarcity of studies that can provide valuable insights about the efficacy of existing interventions, and guidance for developing and implementing effective interventions, especially the aspects of persuasive behavior change component that will motivate the patient to continue with the therapeutical process. Thus, this paper aims to analyze published work on VR and gamification for treating anxiety and depression to determine the effectiveness of current VR interventions in promoting positive mental well-being and preventing these conditions, particularly in adolescents. Additionally, the study aims to identify gaps in current VR treatments and provide guidelines for future research and practice.

To achieve these objectives, the study focuses on addressing the following research questions:

1. What are the types and key features of VR interventions?
2. How effective are the VR interventions in reducing symptoms of anxiety and depression?
3. How is gamification as persuasive technology applied in VR interventions?

In comparing our prior publication (25), “Virtual Reality for Addressing Depression and Anxiety: A Bibliometric Analysis,” and this paper, it's evident that both share a foundational theme of VR interventions for addressing anxiety and depression. The papers diverge in their aims and methodologies, thus presenting unique contributions to the field. The prior bibliometric analysis provided a valuable overview of the research landscape, identifying key trends, prolific authors, and popular journals within the domain of VR for depression and anxiety. In contrast, this paper constitutes a systematic review, offering a deeper comprehension of the efficacy of VR and gamification interventions, specifically in managing anxiety and depression. We meticulously evaluate and synthesize empirical studies, emphasizing clinical outcomes, methodologies, and the impact of gamification, a novel dimension not extensively explored in the earlier work. Our systematic review extends beyond the bibliometric analysis by focusing on the qualitative aspects of the studies, analyzing intervention designs, and assessing the effectiveness of gamification elements. We critically appraise the existing literature, highlighting gaps, methodological approaches, and potential areas for future research. This paper serves to guide practitioners, researchers, and policymakers in making informed decisions regarding the integration and advancement of VR-based interventions together with gamification in anxiety and depression.

## 2. Literature review

VR has emerged as a powerful tool for addressing mental health concerns, particularly through its application in exposure therapy. VR creates immersive and interactive environments that allow individuals to confront anxiety-inducing situations in a safe and controlled manner (26). By utilizing headsets or devices, users can interact with virtual objects and environments, providing a sense of presence and realism. Exposure therapy within VR has shown promising results in treating various mental health conditions. It has been particularly effective in treating phobias and post-traumatic stress disorder (26). Exposure therapy enables individuals to gradually and systematically confront their fears, leading to a reduction in anxiety and avoidance behaviors (27). Virtual environments can be tailored to simulate real-life situations, allowing individuals to develop and practice coping strategies in a controlled setting (19). Moreover, VR can be used to enhance relaxation techniques, mindfulness, and stress reduction. Immersive and interactive experiences provided by VR technology offer unique opportunities for individuals to engage in mindfulness practices and experience stress reduction (9). By leveraging the power of VR, individuals can immerse themselves in tranquil and calming environments, facilitating relaxation, and promoting overall well-being.

Persuasive technology, on the other hand, focuses on designing interactive technologies to influence and persuade individuals' attitudes, behaviors, or beliefs (5). Through persuasive techniques such as feedback, rewards, social influence, and personalization, persuasive technologies aim to motivate users to adopt desired behaviors or make informed decisions (6). By leveraging principles from psychology, social sciences, and human-computer interaction, persuasive technologies can effectively engage users and facilitate positive behavior change. Rewards provide incentives for completing tasks or reaching milestones, personalization and similarity create a comfortable and relatable environment for users while tailoring techniques enable customization based on individual characteristics or preferences (28). An aspect of persuasive technology, gamification, involves applying game design elements and principles to non-game contexts. Gamification has gained attention for its ability to increase user engagement, motivation, and enjoyment (29). By incorporating game-like elements such as challenges, rewards, competition, and progress tracking, gamification aims to enhance participation, learning, and behavior change (30). When applied to mental health interventions, gamification can improve user motivation and adherence to treatment protocols.

Various mental health conditions can benefit from the integration of VR, and persuasive technology. Public speaking anxiety affects a significant percentage of the population, and interventions utilizing exposure therapy and cognitive-behavioral techniques have been effective in addressing this issue (31). Generalized anxiety disorder, panic disorder, social anxiety disorder, and post-traumatic stress disorder are other mental health conditions that can be addressed through exposure therapy and cognitive-behavioral interventions (11, 20, 32).

Public speaking anxiety is a prevalent condition characterized by significant distress while presenting or anticipating speeches in front of an audience (10). Approximately 33% of individuals are estimated to be affected by PSA (10). Exposure therapy and CBT have been recognized as effective interventions for addressing PSA (31, 33).

GAD involves excessive and chronic worry about various aspects of life, such as work, health, or relationships (9). It is estimated that 10% of individuals visiting primary healthcare with mental health disorders suffer from GAD (34). Virtual reality interventions have shown promise in treating GAD by providing immersive and controlled environments for exposure therapy and CBT techniques (9).

Panic disorder is characterized by recurrent and unexpected panic attacks, which manifest as intense episodes of extreme fear or discomfort accompanied by physical symptoms like rapid heartbeat, shortness of breath, and chest pain (32). Exposure to feared conditions, combined with coping skills training, has been identified as a common treatment approach for panic disorders (35). VR interventions can facilitate exposure therapy in a safe and controlled manner, allowing individuals to confront their fears gradually (32).

SAD, also known as social phobia, involves an intense fear of social situations and the fear of being embarrassed, humiliated, or judged by others (11). SAD is a prevalent mental health problem that often leads to avoidance of social interactions and anxiety symptoms when exposed to social situations (36). VR interventions can provide simulated social environments, allowing individuals to engage in exposure therapy and practice social skills in a controlled and supportive setting (11).

PTSD can develop following exposure to traumatic events, such as combat or assault. Individuals with PTSD may experience intrusive memories, nightmares, flashbacks, avoidance of trauma-related triggers, and heightened arousal (20). VR interventions have been utilized to create virtual environments that simulate traumatic events, facilitating exposure therapy and desensitization to trauma-related triggers (20).

VR-CBT is an innovative therapeutic approach that combines VR technology with CBT techniques to address various mental health conditions. Specifically, VRET is a type of VR-CBT that utilizes computer-generated virtual environments to simulate real-life situations, providing individuals with a controlled and safe space to confront and work through their fears, anxieties, or phobias (24, 32).

Exposure therapy, a cognitive-behavioral intervention widely recognized as the “gold standard” for treating anxiety disorders, operates on the principle of gradually exposing individuals to feared situations, enabling them to confront and tolerate their fears, ultimately leading to a reduction in anxiety and avoidance behaviors (27, 33). VRET offers a technological approach to deliver exposure therapy, allowing individuals to engage with situations that may be otherwise difficult to access or control (33). With VRET, therapists can implement individualized, controlled, and gradual exposure therapy that caters to the specific needs of each individual (37).

Mindfulness skills training is an essential component of Dialectical Behavioral Therapy (DBT®) initially developed to address attentional challenges in clinical populations (9). However, individuals with severe symptoms often face difficulties or exhibit reduced motivation to engage in mindfulness practice during training. To overcome these challenges, a new approach called Virtual Reality DBT® Mindfulness Skills Training has been developed. This approach leverages VR technology to facilitate mindfulness learning for individuals with emotion dysregulation who experience attention deficits or limited attentional resources (9).

VRS training refers to the utilization of VR technology as a platform for developing and enhancing social skills in individuals. It involves the creation of simulated social environments and interactions within a VR setting, providing individuals with opportunities to practice and improve their social abilities (21). VRS allows individuals to engage in realistic scenarios that mimic real-life social situations, such as public speaking, interviewing, and interpersonal communication, thus providing a valuable tool for social skills development (38).

## 3. Methods

### 3.1. Search strategy and inclusion criteria

The review was carried out according to the guidelines of Prisma, which governs the procedures for systematic reviews and meta-analyses (39). To locate relevant studies for this review, the following databases were queried for peer-reviewed journal articles: Scopus, Web of Science, and PubMed. The search was conducted on December 20, 2022. When conducting the search, the terms “Virtual reality” or “VR” and “anxiety” or “depression” and “randomised” or “randomized” were used as shown in Table 1.

**Table 1:** Data collection and selection criteria.

Search String	Result	Action (Inclusion)	Action (Exclusion)
TITLE-ABS-KEY ([(“virtual reality” OR “VR”) AND anxiety] OR [(“virtual reality” OR “VR”) AND depression] AND (“randomised” OR “Randomized”))	1,858 document results	Nothing	Nothing
([(“virtual reality” OR “VR”) AND anxiety] OR [(“virtual reality” OR “VR”) AND depression] AND (“randomised” OR “Randomized”)) AND [LIMIT-TO (PUBSTAGE, “final”)] AND [LIMIT-TO (DOCTYPE, “article”)] AND [LIMIT-TO (PUBLICATION DATE, “2014-2022”)] AND [LIMIT-TO (LANGUAGE, “English”)]	942 document results	– Limited to articles in journal, conferences, and book chapters – Limited to articles with status “final”. – Only documents written and published in English language – Limited to articles published between the year 2014 and 2022.	– Excluded documents classified as “editorial”, “Letters”, “Review”, “Conference Review”, “Short Survey”, and Erratum”. – Excluded “Article in Press”. – Documents written and published in other languages such as French, German, Spanish, Chinese, Portuguese, Russian, Hungarian, Italian, and Japanese

### 3.2. Screening criteria

Studies were included if they met the following criteria: randomized controlled trials comparing a VR-enhanced intervention to a control or an active psychological intervention for measuring outcomes related to depression or anxiety, published in peer-reviewed journals, written in English, and published in 2014 or later. This was done because there is evidence that there was a major shift in VR interventions starting in 2014 (25). Studies were excluded from the review if they were case studies, qualitative studies, single-subject designs without control groups/conditions, if the participants did not fulfil the diagnose criteria for anxiety or depression, not written in English, or being peer-reviewed articles. All abstracts were screened by two researchers, and full texts of eligible studies were retrieved. Two researchers then independently examined the full text articles to ensure that those who were included in this systematic review met the pre-established criteria. Any disagreements were resolved through discussion and consultation with a third and fourth author until a consensus was reached.

### 3.3. Included studies

**Figure 1:**
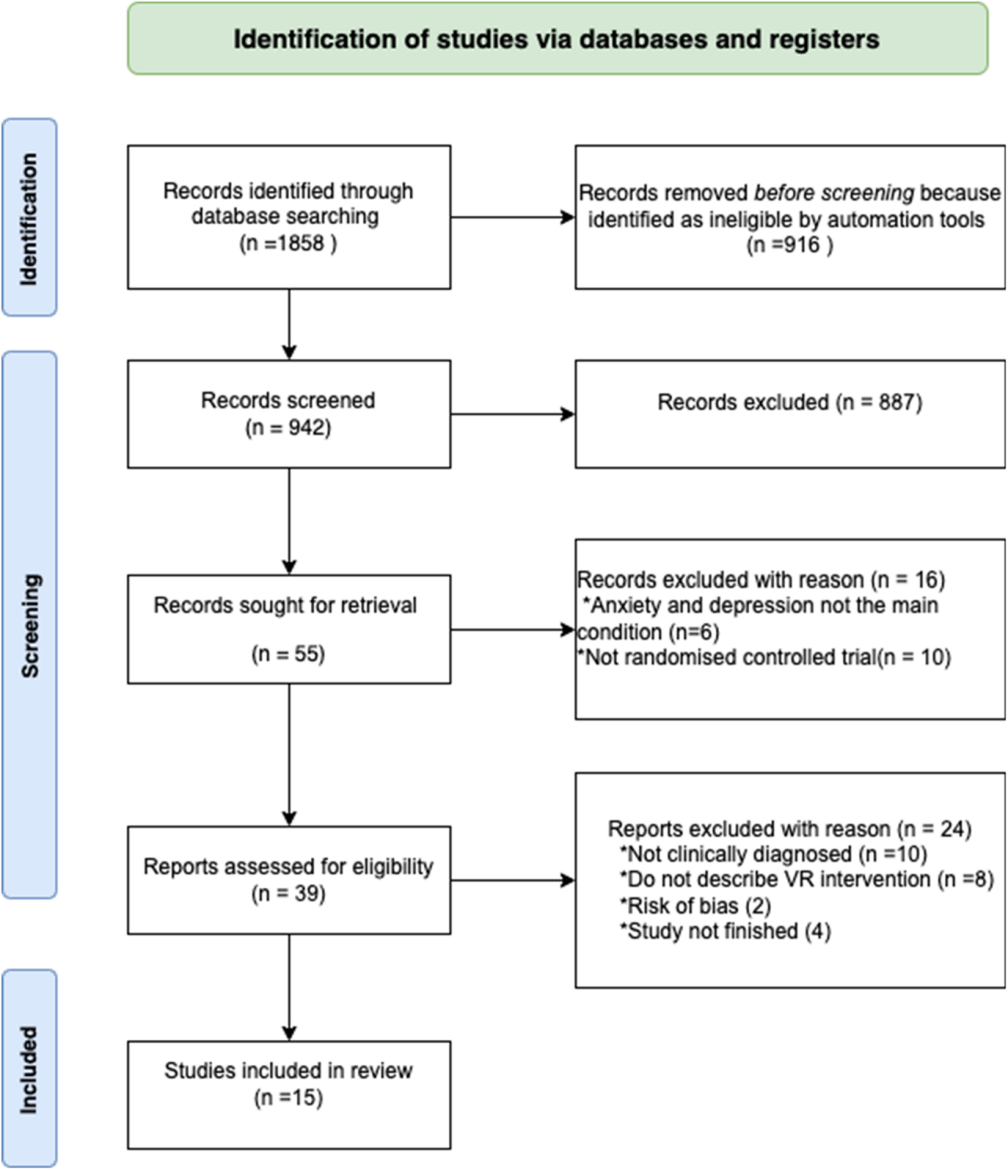
A PRISMA flow diagram showing how data were collected and included in this study analysis.

The Prisma guidelines (39) are well-established protocols for conducting systematic reviews and meta-analyses designed to uphold a rigorous and transparent approach to the review process. In the pursuit of assembling a refined selection of included papers adhering to the PRISMA guidelines, Figure 1 visually elucidates the comprehensive journey undertaken in the paper screening process. The study began by first researcher identifying 1858 records from PubMed, Scopus, and Web of Science. She utilized automated tools were to eliminate 916 records that were deemed as not fitting the criteria. The automated tools employed are the automated filters available in PubMed, Scopus, and Web of Science to refine our paper selection for example the ability to specify only papers in English, randomized controlled trials and publication dates. Out of the remaining 942 records, 887 were further excluded upon screening by all researchers. Researcher one and four then scanned through the 55 reports and they removed 10 records as they were not randomized controlled trials, and 6 more were excluded because they did not focus on anxiety and depression as the main condition. For the remaining processes full text were read to assess their eligibility by researcher one and four. In instances of uncertainty, researcher two and three were consulted for clarification. From the remaining 39 reports, another 10 were eliminated as they did not involve clinically diagnosed patients, and 8 more were excluded as they did not describe the VR interventions used. Additionally, 2 studies were excluded due to concerns regarding their risk of bias and 4 studies were excluded because they were not finished, hence they did not report the results. Ultimately, 15 studies were included in the review (Figure 1). Subsequently, we examined the included papers that met the criteria to identify whether they incorporated gamification features and what those features were.

### 3.3 Screening for gamification features

When we assessed the availability of gamification in the included papers, we considered the following aspects:

1. Engagement and Interaction: We evaluated how the VR application incorporates interactive elements akin to gaming, such as challenges, puzzles, or interactive scenarios that engage users actively.
2. Rewards: We assessed if the VR application offers rewards, points, or badges for completing tasks or achieving milestones related to mental health goals, encouraging users to progress and engage further.
3. Progression and Levels: We checked if the VR application incorporates a sense of progression or levelling up based on the user's achievements, motivating users to continue using the application to reach higher levels.
4. Competition and Social Interaction: We assessed whether the VR application allows users to compete with others, collaborate, or share their progress, fostering a sense of community and encouraging engagement.
5. Customization and Personalization: We assessed if the VR application allows users to customize avatars, environments, or aspects of the experience, providing a personalized and engaging journey.

### 3.5 Quality of included studies

In scientific studies, methodological flaws are relatively common, and these shortcomings may increase the risk of bias in the study results. To minimise the risk of including low-quality studies in our analysis, we utilized a standardized quality index developed by the Cochrane Back Review Group (40). In Table 2, we evaluated each study against the index comprising 12 specific and operationalized questions. Each time a study fulfilled one of these criteria, we awarded it one point. The maximum number of points a study could achieve was 12. If a study had incomplete or missing information for a particular criterion, it received no points for that question. We then tallied the total points achieved, and studies that scored between 0 and 6 were classified as low quality, indicating a relatively high risk of bias. On the other hand, studies that received 7–12 points were classified as high quality and, therefore, at low risk of being affected by bias. All 15 studies we included in our review achieved the required points tally, i.e., 7–12, and were therefore classified as high quality, with a low risk of bias.

**Table 2:** Overall risk of bias.

Author and year	1. Was the method of randomization adequate?	2. Was the treatment allocation concealed?	3. Was the patient blinded to the intervention?	4. Was the care provider blinded to the intervention?	5. Was the outcome assessor blinded to the intervention?	6. Was the drop-out rate described and acceptable?	7. Were all randomized participants analysed in the group to which they were allocated?	8. Are reports of the study free of suggestion of selective outcome reporting?	9. Were the groups similar at baseline regarding the most important prognostic indicators?	10. Were co-interventions avoided or similar?	11. Was the compliance acceptable in all groups?	12. Was the timing of the outcome assessment similar in all groups?	Overall risk of bias
(10)	+	+	?	?	?	+	+	+	+	+	+	+	9
(9)	+	+	−	+	?	+	+	+	+	?	+	+	9
(24)	+	+	−	?	?	+	+	+	+	+	+	+	9
(12)	+	+	−	+	?	+	+	+	+	+	+	?	9
(11)	+	+	−	−	?	+	+	+	+	+	+	+	9
(7, 8)	+	+	?	?	?	+	+	+	+	+	+	+	9
(13)	+	+	−	?	?	+	+	+	+	?	+	+	8
(32)	+	+	−	?	?	+	+	+	+	+	+	?	8
(41)	+	+	−	?	?	+	+	+	+	+	+	+	9
(42)	+	+	−	+	+	+	+	+	?	+	+	+	10
(43)	+	+	?	?	?	+	+	+	+	+	+	?	8
(7, 8)	+	+	?	?	?	+	+	+	+	+	+	+	9
(20)	+	+	−	+	+	+	+	+	+	?	+	?	9
(44)	+	+	−	+	+	+	+	+	+	?	+	?	9
(21)	+	+	−	−	−	+	+	+	+	+	+	?	8

## 4. Results

Table 3 provides a comprehensive overview of the included studies, presenting essential information related to the study's population, the diagnosed condition, the implemented VR intervention, the control group, frequency of the intervention, duration, number of sessions, whether it was conducted individually or in groups, and remission rate. On the other hand, Table 4 focuses on providing information regarding the characteristics of the VR systems employed in the studies. It includes details about the devices used, the scenes incorporated within the VR intervention, whether the VR intervention was delivered remotely or offered self-management features, any inclusion of gamification or persuasive elements, and whether eye tracking and motion tracking mechanisms were implemented.

**Table 3:** Participants and intervention characteristics and intervention parameters.

No	Article	Participants	Condition	VR intervention	Control	Frequency	Min	Number of sessions	Group/Individual	Remission rate
1	(10)	DSM-IV diagnosed; Age: 18–45, *N* = 51	PSA, social anxiety and FNE	VRET	360° Empty, control group	1 time/week &10 weeks after	<4 min	5	Individual	40% remission rate on PSA, 25% remission rate on FNE, and 32% remission rate on social anxiety
2	(9)	DSM-V, Age: 18–65, *N* = 42	GAD	VR-DBT	MBI	1 time/week	10 min	6	Individual	Reduction of anxiety symptoms from Mean of 4.58 to 2.88
3	(24)	DSM-V, Age: 22.5, *N* = 66	GAD	IM-VR-CBT	CBT group	1 time/week	60 min	5	Individual	Reduction of GAD symptoms
4	(12)	DSM-V, Age: 18–53, *N* = 44	SAD	VRET	Waitlist	2 times week	25–30 min	1–8	Individual	88.46% remission rate on SAD
5	(11)	DSM-IV diagnosed, Age: 18–65, *N* = 21	SAD	VRET	AGT	2 times/week	45 min	3	Individual	Reduction of SAD symptoms
6	(7, 8)	DSM-V, Age: 19–30, *N* = 52	SAD	VRS	Control group	4 times/week	No info	8	Individual	reduction on social anxiety and increase in self esteem
7	(13)	Age: 19–23, N1 = 20, N2 = 195	Anxiety and depression	Restorative Environment for relaxation	VR urban environment group, VR park environment group, VR free-roaming group, VR fishing group, and VR watering group	1 time/week	10 min	4	Individual	Reduction of depressive moods of people with mild to moderate anxiety and depression symptoms
8	(32)	DSM-V diagnosed, Age: 35–37, *N* = 54	Panic disorder, anxiety, and depression	VRET	Waitlist	3 times/week	15–30 min	4	Individual	Reduction of anxiety and depression symptoms
9	(41)	GDS-30, Age: >60, *N* = 25	Old-age depression	Relaxation, fitness training, health-promoting education, and psychoeducation	Control group	2 times/week	20 min	4	Group	Reduction of depressive symptoms
10	(42)	DSM-V, BDI-II, Age: 18–70, *N* = 25	PTSD, Depression	Placebo VRET	D-Cycloserine (DCS)	1 time/week	90 min	12	Individual	17.9% remission rate on depression
11	(43)	DSM-V, Age: 50–75, *N* = 77	GAD	VR-Relaxation	VN, VAP	1 time/week	20 min	1	Group	GAD patients showed significantly reduced perceived stress
12	(7, 8)	DSM-V, Age: 19–30, *N* = 52	SAD	VRS	No intervention group	4 times/week	no info	8	Group	Reduced SAD Symptoms
13	(20)	DSM-IV; DSM-IV Axis II, Age: 7–12, *N* = 38	SAD	VRET and VRS	SET-C; SST	2 times/week	60 min	24	Group	60% of children treated with Pegasys-VR™ no longer met diagnostic criteria for SAD
14	(44)	DSM-IV-TR, Age: 18–66, *N* = 60	SAD	VRET	iVET, waiting list.	2 times/week	60 min	5	Individual	Was significantly effective for perceived stress and 47.4% of participants received reliable change
15	(21)	DSM-V diagnosed, Age: 18–50, *N* = 124	SAD	VRS	HCs.	1 time	120 min	1	Individual	Not Clear

**Table 4:** VR intervention, VR system characteristics and gamification.

No	Article	Software and devices	Scenes	Remote/self-management	Gamification/Persuasive technology features	Eye tracking	Motion tracking
1	(10)	360° video-HMD-Samsung Gear VR headset powered by Oculus	The VRS program consisted of 36 social topics total, which could be grouped into 12 situations from three environments. The three environmental contexts were daily life, school life and business life, where each context included four different situations. 1 Small classroom/3 audience members 2 Large classroom/6 audience members 3 Medium conference room/24 audience members 4 Large conference room/approximately 60 audience members.		Progression and levels: At each exposure session, room and audience size increased in-keeping with a traditional graduated hierarchy. They utilized increasing difficulty.	NA	NA
2	(9)	HMD- Oculus Rift DK2 VR goggles, with head tracking.	Beautiful landscapes, floating slowly down a river with trees, boulders and mountains		NA	NA	NA
3	(24)	A non-immersive setting based on the Oculus Rift software	No info	Minimal therapist interaction	NA	NA	NA
4	(12)	360° video VR, HMD-Pico Goblin VR headset.	1. the dinner party. 2.order a drink in front of others without the presence of the party guide. 3.asking the host for directions to the restroom. 4.express dissenting opinions. 5. flag the host to send a drink back. 6.speak to a person of authority.	Virtual therapist	Progression and levels: Increasing level of difficulty		Biometrics, it had the ability to detect voice output and identify head position and movement
5	(11)	360° video VR, HMD-Oculus Rift DKII VR headset with built-in position tracking	1. a small meeting room and 2. large room with Uninterested, Interested, and Neutral audience. Before each speech, the participant was directed to address a specific audience member, focusing specifically on their face. The participant focused on a different (neutral, interested, or uninterested) audience member's face.		Progression and levels: Different levels of difficulty	SMI eye tracker	Position tracking by headset, A HiBall motion-tracking system was used to track head movements.
6	(7, 8)	HMD, consisting of a Samsung Gear VR and a Samsung Galaxy S6.	36 social topics total, which could be grouped into 12 situations from three environments. The three environmental contexts were daily life, school life and business life, where each context included four different situations. Each situation was further specified into three topics, where different topics have different numbers of virtual avatars and various levels of difficulties.	self-led VR training by performing speech tasks as directed by the narrations in the VR content.	Progression and levels: various levels of difficulties. Rewards: The difficulty level increased as the number of virtual avatars became larger.	Eye tracker-Recorded pattens of eyesight movement	Samsung Gear S2 was used to measure the heart rates
7	(13)	360° video, HMD- HTC Vive pro eye, including 1 headset.	Relaxation–two environment types (urban and park) and four interactive activities—automatic viewing, free-roaming, fishing, feeding birds and watering plants in the park environment.		Engagement and interaction: Variation of interactive tasks, similarity to hometown,	Tracked eye movement with headset	EEG and EMG were used to record brain and motion signals.
8	(32)	360° video- VR system was developed on a mobile-based platform. Vega VR software	Digital human avatars or video recordings of real people, various situations (eg, driving a car, taking an elevator, getting on a plane or subway), situations that patients with agoraphobia are afraid of (eg, getting on a plane, driving on bridge).		Progression and levels: use of different levels of exposure and increasing difficulty in each step		
9	(41)	360° video VR. HMD- VR HTC VIVE goggles	Fitness training, relaxation exercises, as well as in health-promoting education and psychoeducation —The garden, weakened and gray at the beginning and becoming more colorful and alive with each session, symbolizes the process of regaining strength and energy.	Self-paced	Rewards, personalisation,	na	na
10	(42)	Head-mounted VR helmet containing two miniature LCD computer screens in front of their eyes.	Jet flying over the towers without crashing and normal city street sounds present. to (e.g., hole in tower, smoke), and auditory effects (e.g., explosion, screaming).	NA	Progression and levels: VR scenarios that gradually increased in detail and intensity to allow for graded hierarchical exposure, Personalisation and customization: Therapist tailored the treatment according to patient's needs.	na	na
11	(43)	Projectors were used to project images at 270°	Landscapes of forests, parks, woods, and rivers generated by machine simulations were projected in the Cave and moved as the participants stepped onto the bicycle	na	na	na	EEG and heart rate were measured.
12	(7, 8)	360° video VR. HMD, Samsung Gear VR powered by Oculus	The VRS content included three environments: school life, business life, and daily life.	Could be performed without therapist help	Progression and levels: have four different levels of difficulty in a way that the number of virtual persons appearing increased. Personalisation and customization: Tailoring speech according to responses.	Recorded eye movement	Speaking time, and heart rate were automatically measured.
13	(20)	Virtual enviroment—A serious game for use on an iPad with internet connection	An entire school, with multiple classrooms, a science lab, cafeteria, gym, hallways, vestibules, lockers, and outside areas, including a playground, snack area, and greenspace native to a school setting and free play engaging in interactions with the virtual characters in the school.	Remotely available on ipad	Engagement and interaction: Included challenges like identifying emotions, arranging comic strips of social interactions, identifying open-ended questions, and fill-in-the-blanks	na	na
14	(44)	nVisor SX HMD	Giving a talk in front of an audience followed by questions from the audience, talking to a stranger, buying, and returning clothes, attending a job interview, being interviewed by journalists, dining in a restaurant with a friend, and having a blind date	na	Personalisation and customization: Tailoring, personalization Progression and levels: levels of difficulty	na	na
15	(21)	HMD- Oculus Rift DK 2 and a joystick	The scenarios provoked an increase of fear and psychophysiological arousal: asking a passenger to release their reserved seat in a virtual train and to cancel a trip in a virtual travel agency.	Pre-recorded instruction. Minimal therapist help	NA	NA	Electromagnetic tracking was used to record the head position of the participants

### 4.1. Participants

Out of the 15 studies that were included, DSM-V was used to diagnose participants in 9 studies, DSM-IV in four studies, and GDS 30 in one study, while the diagnostic tool used was unclear in one study. The age range of participants was from 7 to 75 years, with only one study involving participants under the age of 18. Most studies compared two or more treatments and thus did not have a pure control group, while six studies did have a control group. Among the 12 studies that diagnosed participants with anxiety-related disorders, SAD, GAD, and PTSD were the most commonly diagnosed disorders, whereas only 4 studies diagnosed participants with depression.

### 4.2. VR Intervention

We examined various VR interventions and their characteristics. Out of the 15 studies included, seven focused on VRET (10–12, 20, 32, 42, 44), one on IM-VR-CBT (24), four on VRS (7, 8, 20, 21), one on VR-DTB (9) and three implemented VR intervention aimed at relaxation (13, 41, 43).

### 4.3. Individually/group

The study also examined whether the VR interventions were performed individually or in a group setting. The results showed that 11 studies utilised an individual setting (7–13, 21, 24, 32, 42, 44), while four used a group setting (7, 8, 20, 41, 43).

### 4.4. Control

Regarding control groups, the results showed that two of the studies incorporated some VR into the control group (10, 13). In seven studies, the control group received treatment as usual, such as CBT or PCT (9, 11, 20, 24, 42, 44). Seven studies had a waitlist or no intervention or a control group (7, 8, 10, 12, 32, 41, 44), and one study offered health controls to the control group (21).

### 4.5. Frequency

The results show that the most commonly used frequency in implementing VR interventions was once per week, with 7 out of 15 studies implementing the intervention at this frequency (9, 10, 13, 21, 24, 42, 43). The second highest frequency was twice weekly, used in 5 studies (11, 12, 20, 41, 44). Additionally, there were 3 studies where the VR intervention was implemented only once in a single session (11, 21, 43). One study had a frequency of three times per week (32).

Sessions: In 12 of the studies the VR intervention was done in 4 to 24 sessions (7–10, 12, 13, 20, 24, 32, 41, 42, 44). Three studies had less than 4 sessions (11, 21, 43). The duration per session was reported in 13 studies, with 3 studies offering 10 min or less (9, 10, 32), 4 studies between 15 and 30 min (7, 8, 12, 41, 43), 4 studies between 45 and 60 min ( (11, 20, 24, 44)), and 2 studies did 90 min or more per session (21, 42).

### 4.6. Effectiveness of the study

Twelve studies found a positive treatment outcome, as evidenced by a statistical decrease in symptoms of depression (7–13, 20, 24, 32, 41, 42). However, two studies found that the treatment only had a beneficial effect in reducing perceived stress and not anxiety (43, 44). In one study, it was uncertain whether the intervention had a favorable or unfavorable effect (21).

### 4.7. VR displays

In terms of VR displays, eleven studies implemented the use of HMD (7–13, 21, 41, 42, 44). The results indicate that the most commonly utilized VR headsets are Samsung Gear VR (7, 8, 10) and Oculus Rift (9, 11, 21). Other VR display platforms employed include the Pico VR headset (12), HTC Vive Pro (13, 41), and nVisor SX (44). Additionally, two studies displayed the VR treatment through mobile devices (20, 32), one study employed projectors (43), and one study utilized a VR helmet (42).

### 4.8. Persuasive technology features

Eleven studies implemented gamification and persuasive technology features. Eight studies implemented increasing levels of difficulty (7, 8, 10–12, 32, 42, 44). One study focused on rewards, personalization, and similarity (41), while three studies implemented tailoring (7, 8, 42, 44). Additionally, challenges were incorporated in one study (20).

### 4.9. Remote or self-management features

In 7 out of the 15 studies, the VR interventions implemented self-management features (7, 8, 12, 20, 21, 24, 41). These features were designed to minimize or eliminate the need for therapist involvement during the interventions. One of the studies provided part of the VR intervention for anxiety in children remotely through an iPad (20). The intervention included self-management features that allowed the children to interact with the program independently with the help of their parents. Some of the other studies mentioned are considered to have the potential to be conducted remotely, thanks to self-management features. This means that in these studies, participants could engage with the VR interventions from their locations without needing frequent in-person therapist sessions. Therapists would likely still monitor and support participants as needed, but the intervention could be more flexible and accessible. While therapists were present in these studies, their role primarily focused on ensuring that participants received the intervention as intended.

### 4.10. Eye and motion tracking

Eye or motion movements were tracked and reported in seven studies. Among these, four studies implemented eye tracking through the use of an eye tracker or a VR headset (7, 8, 11, 13, 42). In addition, all seven studies incorporated motion tracking in their methodologies (7, 8, 11–13, 21, 42, 43). In the context of motion tracking, various measurements were taken, including brain activity (43), head movement (11, 12, 21), muscle movements (13), voice detection (7, 8, 12), and heart rate (7, 8, 43).

### 4.11. Scenes used in VR intervention

The findings indicate the presence of four distinct types of scenes across the included studies. These include scenarios designed to expose participants to feared conditions (10–12, 21, 32, 42, 44), visually appealing scenery intended for relaxation and mindfulness (9, 13, 43), scenes created for teaching coping skills specific to diagnosed conditions (7, 8, 41), and games developed to reinforce learned skills (20).

## 5. Discussion

The use of different versions of the DSM-V and DSM-IV for participant diagnosis across studies may introduce variability and potentially affect the comparability of results. It is important to consider the potential impact of using different diagnostic criteria. The age range of participants spanning from 7 to 75 years, indicates a wide demographic representation. However, the findings of this study cannot be generalised to the younger population due to the inclusion of only one study involving participants under 18 (20). Among the diagnosed participants, anxiety-related disorders such as SAD, GAD, and PTSD were the most diagnosed conditions (12, 24, 42). Conversely, only a few studies diagnosed participants with depression, suggesting a potential research gap in understanding the effects of VR and gamification interventions on depression (32, 41, 42).

The majority of the studies included in this review used individual interventions (7–13, 21, 24, 32, 42, 44). The prevalence of individual interventions suggests a recognition of the significance of personalized therapies tailored to individual needs. However, interestingly, the outcomes did not show any significant differences based on whether the interventions were administered individually or in a group setting. This implies that the mode of delivery, whether in a group or individual format, did not impact the treatment outcomes. This finding raises important questions for future studies to explore, particularly in terms of the cost-effectiveness of treatment. If group interventions are as effective as individual interventions, it could have substantial implications for optimizing resources and expanding access to treatment for a larger number of individuals facing similar difficulties.

There was a fairly large difference in the frequency of VR treatment between the studies included in this review, however, this variation did not seem to affect much the outcome of the studies in terms of symptom reduction. Studies that had three or four sessions of VR intervention reported significant symptom reduction (11, 32, 41), as did studies that had many sessions over several weeks (20, 42). The same applies to the duration of the sessions, where studies with 10 min or less/sessions reported significant reductions in symptoms (9, 10), similar to those studies with 60 + min/session (15, 42). This could be seen to suggest that the time of the sessions and the number of sessions is not what determines the effect of the treatment. However, it should be emphasized that there may be other underlying causes, than the number of sessions, or the length of the sessions, that can explain the reduction in symptoms. For example, it could be how the treatment was presented, previous experience with treatment, etc. However, this is something that future research needs to investigate further.

The present study examined various VR interventions and their characteristics, highlighting the versatility of VR technology in addressing different therapeutic objectives. Several types of VR interventions appeared in the reviewed studies: VRET, VR-CBT, VRS, and VR-DBT.

VR-CBT interventions, particularly VRET and VRS, were the most studied, highlighting their prominence and effectiveness in addressing depression and anxiety-related disorders and improving social skills. There were seven interventions on VRET, focusing on exposing individuals to feared conditions in a controlled environment (10–12, 20, 32, 42, 44). Six of the VRET interventions were conducted individually, emphasizing the personalized nature of this approach (10–12, 32, 42, 44). These studies reported positive results in the reduction of anxiety and depression symptoms, with one of them indicating a reduction in perceived stress among patients with GAD and SAD (44). One unique VRET intervention was conducted in a group setting using mobile serious games, which reported a significant decrease in SAD symptoms among 60% of the treated children (20). This highlights the potential of VR technology to deliver effective interventions in a group format. The included VRS studies reported reductions in SAD symptoms and increased self-esteem among the participants, indicating the potential of VR in addressing social anxiety and enhancing self-perception. The IM-VR-CBT reviewed in this study was conducted in an individual setting (24) and it reported reductions in anxiety and depression symptoms, showcasing the effectiveness of combining IM-CBT with VR technology and emphasizing the personalized nature of the approach. The study on VR-DBT reported a reduction in anxiety symptoms. This demonstrates the potential of integrating VR with DBT to target anxiety-related issues. However, the number of studies investigating VR-DTB, and relaxation interventions was relatively limited. Further research in these areas would contribute to a more comprehensive understanding of their potential applications and benefits for anxiety and depression-related disorders. Based on the present study's findings, it is evident that VR interventions have shown promise in addressing various therapeutic objectives. These findings highlight VR interventions' remote usability and potential to complement conventional therapeutic methods, offering a unique and immersive treatment approach. However, it is essential to note that further research is needed to establish the long-term effectiveness and generalizability of VR interventions across different populations and settings.

The majority of the studies have implemented gamification and persuasive technology features in VR interventions specifically targeting anxiety and depression (7, 8, 10–13, 20, 32, 41, 42, 44). These studies have demonstrated positive results in reducing symptoms associated with anxiety and depression. The exact role of gamification and persuasive technology in producing these positive outcomes is not explicitly clear. It is worth noting also that even the studies that did not incorporate gamification features also reported positive results (9, 24). Among the studies, eight of them specifically focused on implementing increasing progression and levels (7, 8, 10–12, 32, 42, 44). This approach involved gradually introducing more challenging tasks or exercises to users over time. The progressive increase in difficulty allows individuals to experience a sense of accomplishment and mastery as they progress through the intervention. This sense of personal growth and improved self-esteem may positively influence anxiety and depression symptoms. The other gamification and persuasive technology features that were emphasized in the studies include rewards, personalization and customization and engagement and interaction (7, 8, 13, 41, 44). Each gamification strategy used may have different results, therefore future study is needed to understand the impact of the gamification strategies. The positive results reported in these studies suggest that incorporating gamification and persuasive technology features into VR interventions can be a valuable strategy for reducing anxiety and depression. By enhancing motivation and engagement, these features contribute to the overall effectiveness of the interventions. However, it is important to note that further research is necessary to explore the long-term effects and applicability of these approaches across different populations and contexts. Continued investigation will provide a deeper understanding of the specific mechanisms through which gamification and persuasive technology impact outcomes in VR-based mental health interventions.

Self-management features in VR interventions are specifically designed to minimize or eliminate the need for direct therapist involvement during the interventions, allowing individuals to take a more independent role in managing their conditions. Although therapists or researchers were still present in the reviewed studies, their role primarily focused on ensuring that participants received the intervention as intended, rather than actively guiding, or delivering the therapy (7, 8). The self-management features embedded in the VR interventions can empower individuals to take control of their own treatment and engage with the intervention at their own pace and convenience. The positive results observed in these studies indicate that individuals were able to effectively engage with VR interventions and derive benefits from them, even in the absence of constant therapist support. VR interventions offer a flexible and accessible approach to the treatment of anxiety and depression, particularly in remote settings where in-person therapy may not be easily accessible or practical. However, it should be noted that the included studies in this review involved researchers, making it challenging to draw definitive conclusions about the efficacy of these interventions in remote scenarios. When considering the remote usage of VR interventions with self-management features, it is crucial to ensure that individuals have adequate support and resources to navigate the intervention effectively. Clear instructions, user-friendly interfaces, and technical support can help individuals make the most of the features and overcome any potential challenges they may encounter (12). Despite the positive results, additional research is needed to fully understand and optimize the remote usability of these interventions in treating anxiety and depression in different populations and settings.

The presence of different types of scenes in the studies included indicates the diversity in approaches and objectives within VR interventions for anxiety and depression. VR Scenes aimed to expose individuals to feared conditions or situations, such as public speaking, social interactions, or phobias has shown promising results in various anxiety-related disorders (10–12, 21, 32, 42, 44). However, ensuring that the VR scenarios accurately represent real-life situations and elicit the intended emotional and physiological responses is important. The research also featured visually appealing scenery scenes designed to promote relaxation and mindfulness. These scenes involved natural environments such as parks, forests, or rivers, aiming to create a calming and immersive experience (9, 13, 43). While these scenes offer individuals an escape from stress and opportunities for relaxation, the extent to which virtual environments can fully replicate the therapeutic benefits of natural surroundings is debatable. Moreover, the research included studies with scenes created for teaching coping skills specific to diagnosed conditions are tailored to address the specific challenges and symptoms of individuals with anxiety and depression disorders (7, 8, 41). It is essential to ensure that these scenes are evidence-based and that the taught coping skills are supported by scientific research. Furthermore, there was one research that featured a game developed to reinforce learned skills. This type of scene can be an engaging and interactive way to consolidate and practice coping strategies and skills acquired during VR interventions (20). However, game-based interventions' efficacy and long-term impact should be further investigated to determine their effectiveness in promoting lasting behaviour change and skill retention.

The results from this study could be highly relevant to the development of the changing healthcare demands, ranging from being personalised to providing remote services that do not require physical interaction (45). Previous research has clearly shown that healthcare needs to adapt to the new demands put on the healthcare system, for instance, be able to deliver treatment on distance (46). This is particularly evident in certain patient groups, e.g., children and adolescents, where gamification has been one way of engaging adolescents (47). Being able to individualise the treatment increases the chances that the treatment will both get the patient to stay in the treatment and that the treatment will have the intended effect (48). This also becomes clear as previous research shows the importance of getting the client to engage in the treatment process (49–51). Since VR technology and gamification could be handy tools to solve many of these issues, like individualising the treatment and giving the treatment at a distance, the need for research in this area is evident.

## 6. Limitations and strengths

We identified several limitations in the reviewed studies. One primary limitation was the homogeneity of participant samples, often comprising of female or male, young adult or university students. This restricts the generalizability of the findings to broader populations and different contexts (10). Additionally, some studies suffered from small sample sizes, which hindered the ability to conduct statistically powerful analyses and direct comparisons between groups (9, 11, 24). It is also important to acknowledge that the review primarily included participants from economically developed countries. This raises a concern regarding external validity, as economic differences may potentially influence the results and limit generalizability to different socioeconomic contexts. Furthermore, it is worth noting that the conclusions that can be drawn from this review are limited due to the relatively small number of studies that fulfilled the inclusion criteria. This highlights the urgent need for additional high-quality research to investigate the topic further and provide a more comprehensive understanding of the subject matter. Another limitation is that the effectiveness of a treatment demonstrated by highly motivated participants within scientific studies does not guarantee the same effectiveness when applied in real-world environments or clinical practice. Treatment success is influenced by various factors, including the level of compliance and collaboration between the psychologist, doctor, and patient (10). Moreover, the issue of patient adherence and motivation emerged as a significant consideration. It has been demonstrated that new approaches are needed to enhance patient adherence and ensure independent implementation of therapy. The lack of encouragement may limit fully self-help approaches to VR therapy (32). Regarding the VR interventions in the reviewed studies, the majority of them were offered VRET, VR-CBT or its variations, such as VR-DBT and VR-MBI. However, other psychotherapeutic approaches that directly impact the subconscious, such as body-oriented therapy and art therapy, have not been extensively explored in the context of VR interventions. This suggests a potential area for future research to investigate the efficacy of these approaches within a VR setting.

Despite the limitations acknowledged, the current study yielded a number of interesting findings. The results of the review indicate that VRET, CBT-VR interventions, VRS, as well as other psychotherapeutic approaches like VR-DBT and VR-MBI, have shown significant improvements in symptoms of GAD, PSA, SAD, anxiety, depression, and difficulties with emotion regulation following treatment (9, 20, 24, 32). Furthermore, the combination of VR and gamification with various psychotherapy methods has resulted in higher treatment retention rates than those who received only traditional psychotherapy. The use of immersive and self-guided VR environments, supported by automated instructions for all aspects of treatment (e.g., treatment rationale, homework), offers increased accessibility for patients, as they can access it anywhere. Incorporating VR and gamification sessions into psychological practice has the potential to enhance the likelihood of achieving remission following treatment.

## 7. Conclusion

This systematic review not only sheds light on the efficacy of VR and gamification in addressing anxiety and depression but also advances our understanding of how multimodality in VR supports and enhances remote working. The findings from this review highlights the potential of diverse sensory and interactive modalities offered by VR not only in anxiety and depression interventions but also in optimising remote work settings. Based on the studies included in this review article, the effect of VR and gamification technology appears to be a good tool for improving mental health. All included studies showed positive effects of the VR and gamification interventions used, regardless of the frequency, intensity or duration of the intervention. However, it should be remembered that there is a large variation between the different VR interventions, which can be seen as a motive for further studies in the area.

We recommended future research to look into ways that address the limitations identified in this review. For example, future research could incorporate more diverse participant samples, ensure larger sample sizes, explore a more comprehensive range of therapeutic approaches within VR and gamification interventions, explore the efficacy VR in remote working and conduct studies in various socioeconomic contexts. By addressing these recommendations, future research can provide more robust evidence on the effectiveness and applicability of VR interventions for anxiety, depression and related disorders in real-world practice.

## Data Availability

The original contributions presented in the study are included in the article/Supplementary Material, further inquiries can be directed to the corresponding author.
